# Improved Non-Grignard Electrolyte Based on Magnesium Borate Trichloride for Rechargeable Magnesium Batteries

**DOI:** 10.1038/s41598-020-64085-2

**Published:** 2020-04-30

**Authors:** Kazuhiko Sato, Goro Mori, Takahiro Kiyosu, Toyonari Yaji, Koji Nakanishi, Toshiaki Ohta, Kuniaki Okamoto, Yuki Orikasa

**Affiliations:** 1 FUJIFILM Wako Pure Chemical Corporation, 1633 Matoba, Kawagoe, Saitama, 350-1101 Japan; 2 SR Center, Ritsumeikan University, 1-1-1 Nojihigashi, Kusatsu, Shiga, 525-8577 Japan; 3 Department of Applied Chemistry, College of Life Sciences, Ritsumeikan University, 1-1-1 Nojihigashi, Kusatsu, Shiga, 525-8577 Japan; 4 Present Address: Laboratory of Advanced Science and Technology for Industry, University of Hyogo, 3-1-2 Koto, Kamigori-cho, Ako-gun, Hyogo, 678-1205 Japan

**Keywords:** Batteries, Batteries

## Abstract

The high anodic stability of electrolytes for rechargeable magnesium batteries enables the use of new positive electrodes, which can contribute to an increase in energy density. In this study, novel Ph_3_COMgCl-, Ph_3_SiOMgCl-, and B(OMgCl)_3_-based electrolytes were prepared with AlCl_3_ in triglyme. The Ph_3_COMgCl-based electrolyte showed anodic stability over 3.0 V vs. Mg but was chemically unstable, whereas the Ph_3_SiOMgCl-based electrolyte was chemically stable but featured lower anodic stability than the Ph_3_COMgCl-based electrolyte. Advantageously, the B(OMgCl)_3_-based electrolyte showed both anodic stability over 3.0 V vs. Mg (possibly due to the Lewis acidic nature of B in B(OMgCl)_3_) and chemical stability (possibly due to the hard acid character of B(OMgCl)_3_). B(OMgCl)_3_, which was prepared by reacting boric acid with a Grignard reagent, was characterized by nuclear magnetic resonance (NMR) spectroscopy, Fourier-transform infrared spectroscopy (FTIR), and X-ray absorption spectroscopy (XAS). The above analyses showed that B(OMgCl)_3_ has a complex structure featuring coordinated tetrahydrofuran molecules. ^27^Al NMR spectroscopy and Al *K*-edge XAS showed that when B(OMgCl)_3_ was present in the electrolyte, AlCl_3_ and AlCl_2_^+^ species were converted to AlCl_4_^−^. Mg *K*-edge XAS showed that the Mg species in B(OMgCl)_3_-based electrolytes are electrochemically positive. As a rechargeable magnesium battery, the full cell using the B(OMgCl)_3_-based electrolyte and a Mo_6_S_8_ Chevrel phase cathode showed stable charge-discharge cycles. Thus, B(OMgCl)_3_-based electrolytes, the anodic stability of which can be increased to ~3 V by the use of appropriate battery materials, are well suited for the development of practical Mg battery cathodes.

## Introduction

Rechargeable magnesium batteries are promising alternatives to lithium-ion batteries, featuring high theoretical volumetric capacity (3832 mAh/cm^3^), high safety derived from the nondendritic behavior of deposited magnesium, and the high abundance of magnesium metal compared with that of lithium^1^. However, the advantages of magnesium batteries are only possible if appropriate electrolytes and cathodes are used with magnesium metal. Although various electrolytes have been developed for magnesium batteries by several pioneering researchers, they require further improvement for practical application^2^.

Early studies have shown that plating/stripping of magnesium does not occur in the electrolytes of simple magnesium salts, such as Mg(ClO_4_)_2_, in conventional organic solvents owing to the passivating surface/blocking layer of magnesium^2,3^. Aurbach *et al*. reported a prototype magnesium battery comprising Mg metal anodes and a Chevrel phase cathode^4^. The “dichloro complex” or “all-phenyl complex” electrolyte, which is a complex solution composed of organomagnesium compounds or organomagnesium halide with a Lewis acid such as AlCl_3_, was used for their prototype batteries^4–7^. Non-Grignard magnesium-based electrolytes, such as amide-^8,9^, phenolate-^10,11^, and alkoxide-based^12–15^ ones, have also been developed to be used in combination with active materials such as sulfur and to decrease sensitivity to air/moisture.

For example, Kim *et al*. developed [Mg_2_(μ-Cl)_3_·6THF][HMDSAlCl_3_] (THF = tetrahydrofuran, HMDS = hexamethyldisilazane) and used it in a Mg/S battery^9^. Doe *et al*. reported a magnesium aluminum chloride complex (MACC) and showed that this electrolyte, comprising fully inorganic salts, can be used in a magnesium battery^16,17^. In recent years, it has also been reported that even an electrolyte without a Lewis acid causes magnesium plating/stripping. For example, Mg(TFSI)_2_/triglyme (TFSI = bistriflimide) showed high electrochemical stability toward oxidation and can cause reversible magnesium plating/stripping^18–20^. A carborane-based electrolyte without a Lewis acid was also reported to exhibit high Coulombic efficiency and high anodic stability for SUS and Al electrodes^21–24^.

Magnesium is easily corroded by AlCl_3_ solution in thionyl chloride, although this corrosion can be suppressed by a solution of Mg(AlCl_4_)_2_ in thionyl chloride^25^. The Coulombic efficiency of magnesium plating/stripping can be improved by conditioning of the MACC electrolyte in THF^26,27^. Moreover, magnesium plating/stripping occurs even in the monoglyme-MACC electrolyte, the Coulombic efficiency and open circuit potential (OCP) of which differ from those of the same electrolyte in THF^17,27^. The addition of MgCl_2_ to Mg(TFSI)_2_ in 1,2-dimethoxyethane significantly improved the electrochemical performance in terms of reversible magnesium deposition^28^. In this case, PF_6_^–^ anions passivate the Mg anodes, although reversible Mg deposition/dissolution commence via the addition of either MgCl_2_ or LiCl^29^. However, it is not yet known which of these electrolytes affect the deposition/dissolution of magnesium and anodic stability.

The development of magnesium batteries requires magnesium electrolytes to show high anodic stability, be safe and easy to handle, and allow reversible magnesium plating and stripping. From the viewpoint of safety, it is desirable for the magnesium electrolyte to contain species that impart low flammability, low reactivity with ambient air, and other such properties^30^. In addition, previous reports suggest that electrolytes containing an inorganic anion show higher anodic stability than those containing alkyl or alkoxide anions^23,24,30^. Therefore, this study examined magnesium salts with an inorganic anion, borate, as magnesium electrolytes. Three novel magnesium salts, Ph_3_COMgCl, Ph_3_SiOMgCl, and B(OMgCl)_3_, in triglyme and AlCl_3_ solvents were synthesized and investigated by cyclic voltammetry (CV) and linear sweep voltammetry (LSV). In addition to electrochemical performance, chemical stability was discussed on the basis of nuclear magnetic resonance (NMR) measurements. The B(OMgCl)_3_-based electrolyte, which showed high anodic stability, was further studied by NMR spectroscopy, Fourier-transform infrared (FTIR) spectroscopy, and X-ray absorption spectroscopy (XAS).

## Experimental Section

### Preparation of electrolytes

**Figure 1:**
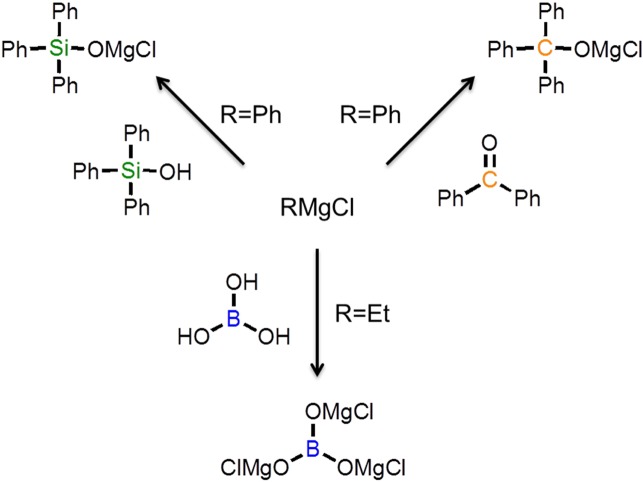
Schematic synthetic routes of various magnesium salts used in this study.

The magnesium salts, Ph_3_COMgCl, Ph_3_SiOMgCl, and B(OMgCl)_3_, were prepared by reacting benzophenone, triphenylsilanol, and boric acid, respectively, with a Grignard reagent. The schematic synthetic routes of the magnesium salts used in this study are shown in Fig. 1. The detailed procedures are described in the Supporting Information. ^1^H NMR measurements confirmed the disappearance of the O–H proton of the reactant. Titration indicated that the ratio of magnesium and chlorine coordinated to THF was Mg:Cl = 1:1 for all magnesium salts. In an argon-filled glove box, each magnesium salt in triglyme was heated at 40 °C. Aluminum chloride was added, and the reaction mixture was stirred and then cooled to room temperature. The preparation conditions are shown in Table S1.

### Characterization

^1^H, ^11^B, and ^27^Al NMR spectra were recorded on a Fourier-transform NMR spectrometer (400 MHz, JMN-ECS400, JEOL). The ^1^H NMR spectrum of B(OMgCl)_3_ was measured using a filtered solution of the sample in DMSO-*d*_6_. The ^27^Al NMR spectra of AlCl_3_/triglyme, B(OMgCl)_3_-AlCl_3_/triglyme, AlCl_3_/triglyme-THF, and B(OMgCl)_3_-AlCl_3_/triglyme-THF were measured using neat samples. The ^11^B NMR spectra of B(OH)_3_ and B(OMgCl)_3_ were measured using samples dissolved in CD_3_OD/CD_3_CO_3_D mixed solvent (50/50 vol%). The ^11^B NMR spectrum of B(OMgCl)_3_-AlCl_3_/triglyme was measured using a sample diluted with THF-*d*_8_. Attenuated total reflection FTIR spectroscopy was performed using a Fourier-transform infrared spectrometer (Nicolet 380, Thermo Scientific). Powder X-ray diffraction (XRD) patterns were measured in the range of 2*θ* = 5–50 ° using a powder X-ray diffractometer (Miniflex 600, Rigaku) operated at a 40 kV voltage and 15 mA current using Cu Kα radiation (λ = 1.5406 Å). XAS measurements of the liquid electrolyte were performed at beamline BL-10 of the SR Center of Ritsumeikan University (Japan). A home-made measurement cell, where the electrolytes are separated from vacuum by a silicon nitride window, was used^31^. Mg and Al *K*-edge XAS spectra were measured in fluorescence mode using a silicon drift detector.

### Electrochemical measurements

CV and LSV were performed in an argon-filled glovebox (<0.1 ppm each of water and oxygen) using an electrochemical measurement system (VMP3, Bio-Logic Science Instruments). CV measurements were conducted within the potential range of −1.0 to 3.5 V vs. Mg at a scan rate of 5 mV/s using a three-electrode cell. This cell has Pt (diameter (*φ*): 3.0 mm, disk, BAS Co., Ltd.), Mg (*φ*: 1.6 mm, rod, 99.95%, Nilaco Co., Ltd.), and Mg (*φ*: 1.6 mm, rod, 99.95%, Nilaco Co., Ltd.) as the working, reference, and counter electrodes, respectively, immersed in 2 mL of electrolyte. LSV measurements were conducted within the potential range of OCP to 3.5 V vs. Mg at a scan rate of 5 mV/s using the same cell, but with various working electrodes. These working electrodes include Pt (*φ*: 3.0 mm, disk, BAS Co., Ltd.), glassy carbon (GC; *φ*: 3.0 mm, disk, BAS Co., Ltd.), Al (thickness: 0.1 mm, plate, 99.999%, Nilaco Co., Ltd.), SUS304 (thickness: 0.2 mm, plate, Nilaco Co., Ltd.), and Mo (*φ*: 1.5 mm, rod, 99.95%, Nilaco Co., Ltd.).

The galvanostatic deposition of magnesium was carried out in a three-electrode cell under a current density of 1 mA/cm^2^ applied to the Pt working electrode for 50 h. Scanning electron microscopy (SEM) was performed using a field emission scanning electron microscope (S-4800, Hitachi, Ltd.) to observe the precipitate on the electrode. Synchrotron XRD measurements of plated magnesium were performed at beamline BL5S2 of the Aichi Synchrotron Radiation Center (Japan). A glass capillary with an outer diameter of 0.3 mm was filled with the sample and sealed by a resin in an argon-filled glove box. XRD data were collected using a Debye-Scherrer optical system with a two-dimensional semiconductor detector (PILATUS 100 K, Dectris). The wavelength of the X-ray was calibrated to *λ* = 0.6995 Å.

### Battery testing

Cu_2_Mo_6_S_7.8_ was purchased from Nippon Inorganic Colour & Chemical Co., Ltd. Hydrochloric acid and ultrapure water were purchased from FUJIFILM Wako Pure Chemical Corporation and used as received. Mo_6_S_8_ Chevrel phase, the active material, was prepared by chemical leaching of Cu_2_Mo_6_S_7.8_ in HCl/H_2_O with oxygen bubbling. The electrode slurry consisted of 80 wt% active material, 10 wt% acetylene black (DENKA BLACK, Denka Co., Ltd.), and 10 wt% polyvinylidene fluoride (KF Polymer L#7305, Kureha Corporation) dissolved in 1-methyl-2-pyrrolidone using a planetary ball mill. The slurry was coated onto carbon paper, which served as the current collector, and dried under vacuum at 80 °C for 1 h and subsequently at 120 °C for 5 h. It was reported that carbon electrode is stable during magnesium charge-discharge^32^. The resulting sheet, along with those of AZ31 (thickness: 0.5 mm, Izumi Metal Corporation) and glass fiber filter (GA100, Advantec), were cut into disks 16 mm in diameter to form the cathode, anode, and separator, respectively. The test cell was prepared by laminating the cathode, separator, and AZ31 anode on a CR2032 coin-type cell (Hohsen Corp.), which was then filled with electrolyte. Galvanostatic charge-discharge tests were carried out at a constant current of C/50 rate and temperature of 25 °C using a charge/discharge unit (ABE 1024-05R1, Electrofield). The experimental conditions were based on the theoretical capacity of Mo_6_S_8_ (128 mAh/g) in the range of 0.5–1.9 V vs. Mg at 25 °C.

## Results and Discussion

**Figure 2:**
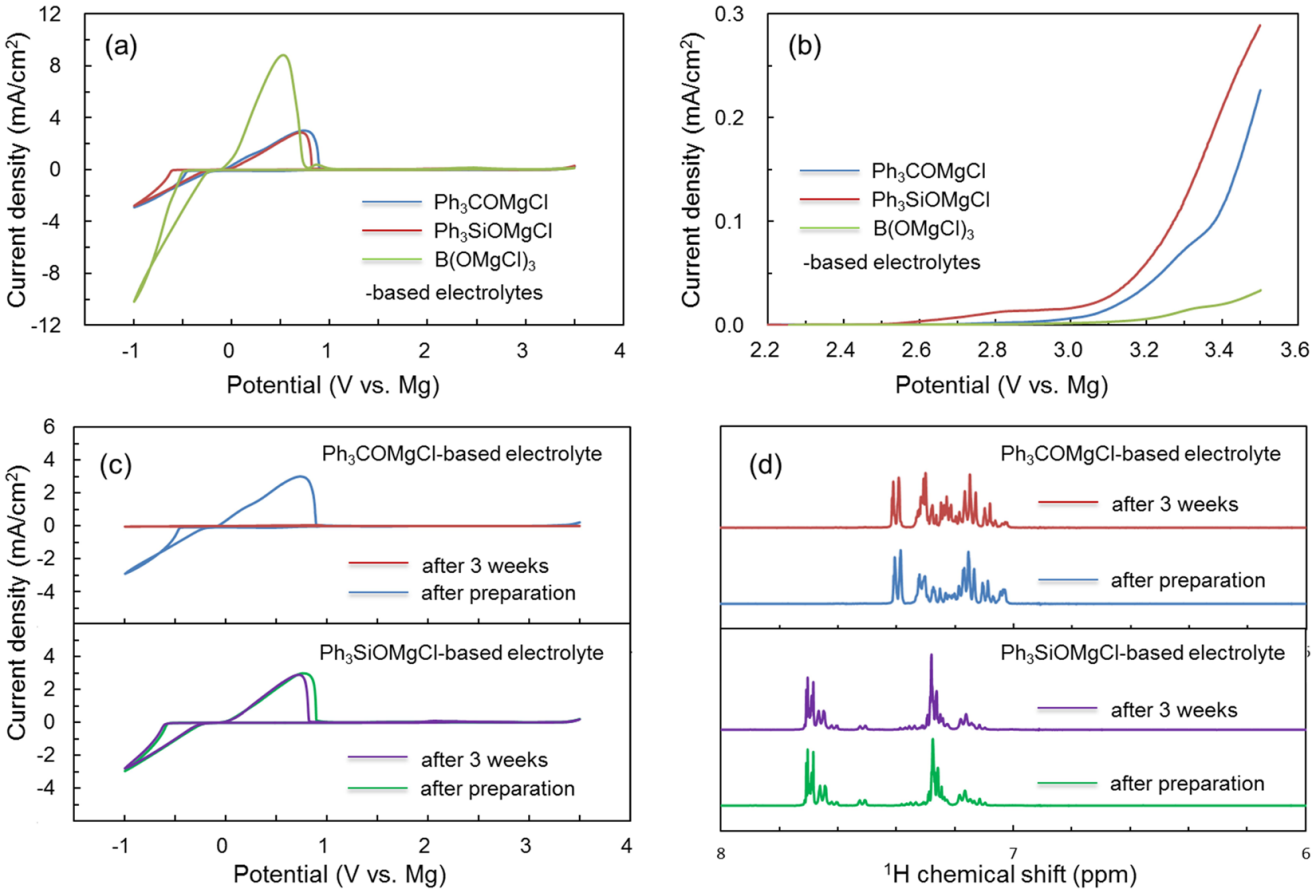
(**a**) Cyclic voltammograms of Ph_3_COMgCl- (blue), Ph_3_SiOMgCl- (red), and B(OMgCl)_3_-based (green) electrolytes at the 100^th^ cycle. Measurements were taken at a scan rate of 5 mV/s at 20 °C using Pt, Mg, and Mg as the working, reference, and counter electrodes, respectively, in a three-electrode cell. (**b**) Linear sweep voltammograms of Ph_3_COMgCl- (blue), Ph_3_SiOMgCl- (red), and B(OMgCl)_3_-based (green) electrolytes at a scan rate of 5 mV/s at 20 °C. (**c**) Cyclic voltammograms at the 100^th^ cycle measured at a scan rate of 5 mV/s at 20 °C. The blue and red curves represent the first measurement and measurement after three weeks, respectively, for the Ph_3_COMgCl-based electrolyte. The green and purple curves represent the first measurement and measurement after three weeks, respectively, for the Ph_3_SiOMgCl-based electrolyte. (**d**) ^1^H NMR spectra of the Ph_3_COMgCl-based electrolyte as prepared (blue) and three weeks after preparation (red) and Ph_3_SiOMgCl-based electrolyte as prepared (green) and three weeks after preparation (purple).

We first examined the electrochemical behaviors of the Ph_3_COMgCl-, Ph_3_SiOMgCl-, and B(OMgCl)_3_-based electrolytes. Figure 2(a) displays the cyclic voltammograms of the electrolytes, prepared by mixing a magnesium salt with AlCl_3_ in triglyme, at the 100^th^ cycle. They show reversible cathodic and anodic currents with Mg plating and stripping at 0 V vs. Mg. The B(OMgCl)_3_-based electrolyte shows the highest reversible current density for Mg plating and stripping. The cyclic voltammograms of the Ph_3_COMgCl- and Ph_3_SiOMgCl-based electrolytes, consisting of magnesium salts of similar structure, exhibit similar shapes with almost the same current densities. The Coulombic efficiencies are 75.7%, 81.5%, and 64.0% for the Ph_3_COMgCl-, Ph_3_SiOMgCl-, and B(OMgCl)_3_-based electrolytes, respectively. The three electrolytes have different anodic stabilities. Figure 2(b) displays the linear sweep voltammograms of the electrolytes. The linear sweep voltammogram of the Ph_3_COMgCl-based electrolyte shows anodic stability close to approximately 3.0 V vs. Mg. On the other hand, for the Ph_3_SiOMgCl-based electrolyte, a weak oxidation current is observed at 2.6 V, and the anodic current increases at about 3.0 V, which is close to the decomposition potential of the Ph_3_COMgCl-based electrolyte. Comparison of the linear sweep voltammograms of the Ph_3_COMgCl- and Ph_3_SiOMgCl-based electrolytes shows that the central element influences anodic stability. Among these electrolytes, the B(OMgCl)_3_-based electrolyte shows the highest anodic stability over 3.0 V. The B(OMgCl)_3_-based electrolyte also exhibits long-term stability. Figure 2(c) shows the cyclic voltammograms of the Ph_3_COMgCl- and Ph_3_SiOMgCl-based electrolytes measured three weeks after the first measurement in the presence of 0.1 ppm each of H_2_O and O_2_ in an argon-filled glove box. The Ph_3_COMgCl-based electrolyte shows no current associated with magnesium plating/striping, while the Ph_3_SiOMgCl-based electrolyte shows almost the same CV curve even after three weeks.

The difference in stability is related to the chemical structures of the electrolytes. Figure 2(d) shows the ^1^H NMR spectra of the Ph_3_COMgCl- and Ph_3_SiOMgCl-based electrolytes as prepared and after three weeks. The ^1^H NMR spectrum of the Ph_3_COMgCl-based electrolyte from 7.20 to 7.35 ppm changes between after preparation (blue) and after three weeks (red), which suggests the structural change of the Ph_3_CO^–^ anion during preservation in the glove box. This chemical instability is hypothesized to be the cause of the change in the cyclic voltammogram of the Ph_3_COMgCl-based electrolyte in Fig. 2(c). On the other hand, the Ph_3_SiOMgCl-based electrolyte shows almost the same ^1^H NMR spectrum as prepared (green) and after three weeks (purple), which indicates that the initial structure of the Ph_3_SiO^–^ anion is maintained. Although the difference between the magnesium salts used in the electrolytes is only a single element, the Ph_3_SiOMgCl-based electrolyte shows lower anodic stability and higher chemical stability compared with the Ph_3_COMgCl-based electrolyte. The shape of the CV curve of the B(OMgCl)_3_-based electrolyte is nearly the same even after three weeks, which suggests the chemical stability of the BO_3_^3−^ anion structure (Figure S1 in the Supporting Information). Chemical stability may be related to the strengths of the Si–O, B–O, and C–O bonds.

Density functional theory calculations suggest that chemical stability is related to the strength of C–O, Si–O, and B–O bonds of anions determined using the hard and soft acids and bases principle (Supporting Information), further implying that the C–O bond of Ph_3_COMgCl-based electrolytes can be weakened by Ph substitution (Supporting Information). Moreover, according to previous reports, the Lewis acidic character of B(OMgCl)_3_ may enhance the anodic stability of the electrolyte^7,15,33,34^. Interestingly, elemental differences in the structure can change electrolyte properties, such as anodic stability and chemical stability. The B(OMgCl)_3_-based electrolyte shows both excellent anodic stability and chemical stability despite its low Coulombic efficiency. Therefore, we will discuss this electrolyte in more detail.

**Figure 3:**
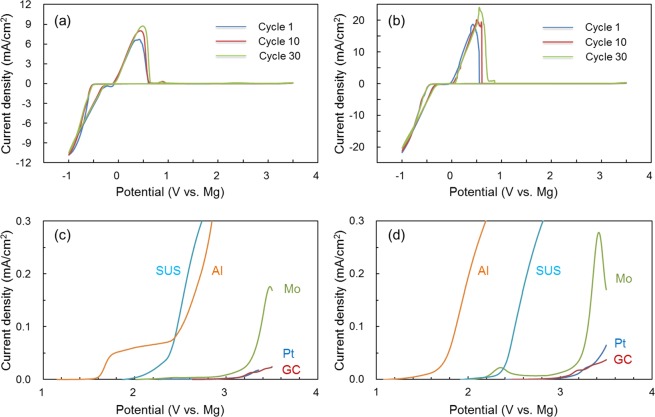
Cyclic voltammograms of the B(OMgCl)_3_-based electrolyte in (**a**) triglyme and (**b**) triglyme-THF measured at a scan rate of 5 mV/s at 20 °C. Linear sweep voltammograms of the B(OMgCl)_3_-based electrolyte with Pt (blue), glassy carbon (red), Mo (green), SUS (light blue), and Al (orange) electrodes in (**c**) triglyme and (**d**) triglyme-THF measured at a scan rate of 5 mV/s.

The cyclic voltammograms of the B(OMgCl)_3_-based electrolyte in triglyme and triglyme-THF are shown in Fig. 3(a,b), respectively. Cathodic and anodic currents around 0 V vs. Mg appear repeatedly in each cycle, which suggests that magnesium metal reversibly plates and strips on Pt within the CV measurement range. The B(OMgCl)_3_-based electrolyte in triglyme shows an overpotential of approximately 570 mV at 1.0 mA/cm^2^. Coulombic efficiency improves from 38.2% at the first cycle to 57.6% at the 30^th^ cycle. However, it is 64.0% even at the 100^th^ cycle, which indicates that the Coulombic efficiency of the B(OMgCl)_3_-based electrolyte in triglyme is not improved by the conditioning process as previously reported^19,20^. As shown in Fig. 3(b), the use of a triglyme-THF mixed solvent results in improved current density, probably because THF has a lower viscosity than triglyme^35,36^. The overpotential of the B(OMgCl)_3_-based electrolyte in triglyme-THF is approximately 490 mV at 1.0 mA/cm^2^. The conductivity of the B(OMgCl)_3_-based electrolyte at 27 °C improves from 2.15 mS/cm without THF to 3.8 mS/cm with THF. Thus, the current density is enhanced by adding THF. The Coulombic efficiency of the B(OMgCl)_3_-based electrolyte increases from 43.4% at the first cycle to 62.8% at the 30^th^ cycle. The cycle dependency of Coulombic efficiency is illustrated in Figure S2 in the Supporting Information. The origin of the insufficient overvoltage and Coulombic efficiency will be discussed after the characterization of the electrolyte.

Figure 3(c,d) show the linear sweep voltammograms of the B(OMgCl)_3_-based electrolyte in triglyme and triglyme-THF, respectively. The B(OMgCl)_3_-based electrolyte in triglyme shows anodic stability over 3.0 V vs. Mg using Pt and GC. However, its anodic stability decreases to 1.6 V using Al and 2.0 V using SUS. This behavior is also observed in the B(OMgCl)_3_/triglyme-THF electrolyte. The trend in the anodic stability of the B(OMgCl)_3_-based electrolyte is Al < SUS < Pt, GC regardless of the solvent. The same trend is observed in bisamide- and dialkoxide-based electrolytes, which indicates that Cl^–^ has an oxidatively unfavorable effect on Al and SUS^8,13^. Using Mo, the B(OMgCl)_3_-based electrolyte in triglyme shows an anodic stability of about 3.0 V, while the B(OMgCl)_3_-based electrolyte in triglyme-THF shows a weak decomposition current from 2.0 V and increased current at about 3.0 V vs. Mg. Pt and GC can be used as current collectors with the B(OMgCl)_3_-based electrolyte up to 3.0 V vs. Mg, and SUS up to about 2.0 V vs. Mg. Mo can be used with the B(OMgCl)_3_-based electrolyte in triglyme up to approximately 3.0 V vs. Mg.

**Figure 4:**
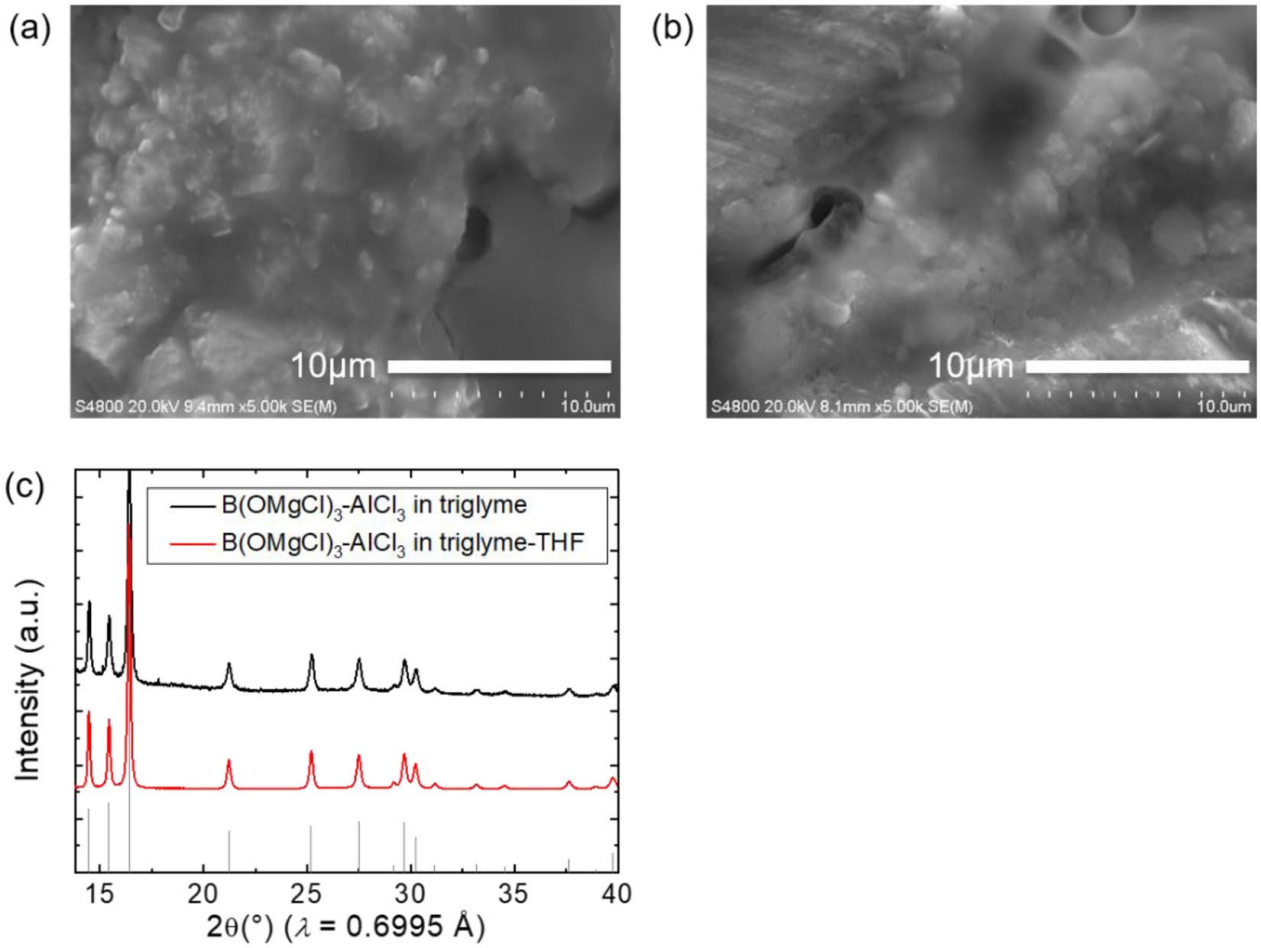
SEM images of electrodeposited Mg from the B(OMgCl)_3_-based electrolyte in (**a**) triglyme and (**b**) triglyme-THF on a Pt electrode. (**c**) Synchrotron radiation XRD patterns of electrodeposited Mg from the B(OMgCl)_3_-based electrolyte in triglyme (black) and triglyme-THF (red) compared with that of reference Mg (gray).

We confirmed that the plated product is Mg metal without dendritic formation. SEM images of plated magnesium from the B(OMgCl)_3_-based electrolyte in triglyme and triglyme-THF on Pt are shown in Fig. 4(a,b), respectively. The SEM images show that the magnesium from the B(OMgCl)_3_-based electrolytes in different solvents appears to co-exist with a few grain-like particles and flat surface. The products from either electrolyte do not show dendritic morphology. The magnesium deposited from both electrolytes does not have a well-defined crystal morphology, and its grain size appears to be smaller than 2 μm. The crystalline edges are not clear, probably caused by the long-term reduction during sample preparation. Density functional theory calculations revealed the cathodic instability of Mg-coordinated glyme electrolytes^37^. The long-term reduction causes partial decomposition of the B(OMgCl)_3_-based electrolyte, resulting in the mossy structure observed in the SEM images. Synchrotron radiation XRD measurement confirmed that the precipitate is magnesium, as shown in Fig. 4(c). Both XRD patterns for the B(OMgCl)_3_-based electrolytes with/without THF show the peaks at the same position as those of the reference for Mg, although the scattering pattern in the (002) plane at 2θ = 15.4° is slightly smaller. Previous reports suggest that a small reflection of the (002) plane for magnesium represents a small grain size^29,38–40^. Plated magnesium can become polycrystalline depending on the precipitation conditions, and the change appears in the (002) plane, which is the close-packed surface of the magnesium crystal^39^. The small grain size of plated magnesium from the B(OMgCl)_3_-based electrolytes is considered to be caused by the influence of such polycrystallization.

**Figure 5:**
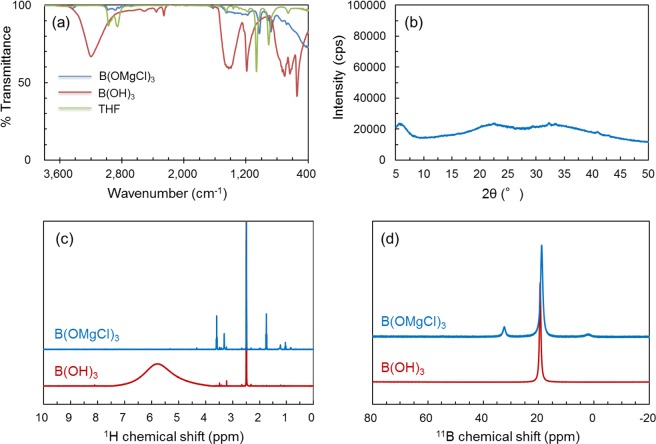
(**a**) Comparison of the FTIR transmittance spectra of B(OH)_3_ (red), B(OMgCl)_3_ (blue), and THF (green) from 400 to 3800 cm^–1^. (**b**) Powder XRD pattern of B(OMgCl)_3_. (**c**) ^1^H NMR spectra of B(OMgCl)_3_ filtrate with DMSO-*d*_6_ (blue) and B(OH)_3_ dissolved in DMSO-*d*_6_ (red). (**d**) ^11^B NMR spectra of B(OMgCl)_3_ (blue) and B(OH)_3_ (red) dissolved in CD_3_OD/CD_3_CO_2_D (50/50 vol%).

Although Mg plating and stripping with high anodic stability is observed, the Coulombic efficiency is approximately 60% at the 100^th^ cycle. We will discuss the possibility of this Coulombic efficiency considering the chemical structure of the B(OMgCl)_3_-based electrolytes inferred from FTIR, NMR, and X-ray absorption fine structure (XAFS) analyses. First, we rule out the possibility of residual B(OH)_3_. FTIR measurements of the B(OMgCl)_3_ product, B(OH)_3_ reactant, and THF solvent are shown in Fig. 5(a). While the absorption related to the *γ*_O–H_ peak of B(OH)_3_ (red) at about 3200 cm^−1^ is not observed in the spectrum of B(OMgCl)_3_, absorptions with similar shape to those of THF (green) are observed in the range of 800–1100 and 2800–3100 cm^−1^. The absorption at the latter is attributed to the *ν*_C–H_ of THF and those at 877 and 1026 cm^−1^ are attributed to the red-shifts of *δ*_C–C–O_ (905 cm^–1^) and *γ*_C–O_ (1065 cm^−1^) of THF, respectively^41,42^. These results imply the consumption of the B(OH)_3_ reactant, which is also supported by the absence of the associated peak in the XRD pattern of the synthesized B(OMgCl)_3_ shown in Fig. 5(b). The absence of diffraction peaks suggests that the B(OMgCl)_3_ salt has a disordered molecular arrangement and no other magnesium salt such as MgCl_2_ is formed. The ^1^H NMR spectra of B(OMgCl)_3_ and B(OH)_3_ dissolved in DMSO-*d*_6_ are shown in Fig. 5(c). The broad O–H peak at 5.8 ppm detected in B(OH)_3_ is not observed in B(OMgCl)_3_, which further proves that B(OH)_3_ is completely consumed in the reaction, as implied by the FTIR and XRD results. The new peaks observed at 1.76 and 3.59 ppm indicate that THF remains in the synthesized magnesium salt^43^, which is also consistent with the FTIR results showing the coordination of THF to the magnesium salt.

Although the B(OH)_3_ itself no longer remains, the presence of the BO_3_^3−^ unit is confirmed by the ^11^B NMR spectra of B(OH)_3_ and B(OMgCl)_3_, which were both dissolved in CD_3_OD/CD_3_CO_2_D (50/50 vol%) (Fig. 5(d)). The main peak in the ^11^B NMR spectrum of B(OMgCl)_3_ is located at around 19 ppm, which is almost the same as that of B(OH)_3_^44^. The presence of this peak indicates that the BO_3_^3−^ unit remains in the structure of B(OMgCl)_3_. The origin of the other peaks (around 2 and 32.4 ppm) are unknown at present. However, these peaks are unrelated to the solvents used for the measurement (CD_3_OD and CD_3_CO_2_D) because they are also detected in the B(OMgCl)_3_-based electrolytes described later.

**Figure 6:**
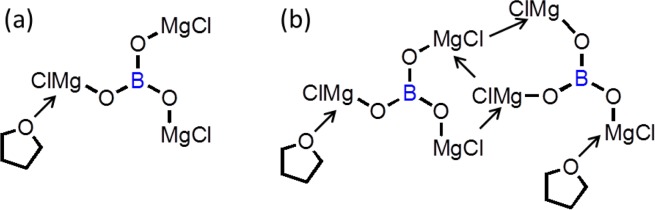
Plausible structures of the product from boric acid and ethylmagnesium chloride.

B(OH)_3_ is not detected in the FTIR, XRD, and ^1^H NMR measurements, and the BO_3_^3−^ unit remains in the B(OMgCl)_3_ salt according to the ^11^B NMR measurement. Moreover, the molar ratio of B(OMgCl)_3_, measured by inductively coupled plasma-atomic emission spectroscopy and Cl titration, is B:Mg:Cl = 1:3:3. These results indicate that the Grignard reagent mainly reacts with B(OH)_3_ as a Brønsted acid to form B(OMgCl)_3_. B(OMgCl)_3_ does not show the ordered arrangement and disproportionated structures observed in the XRD measurement. In addition, the FTIR and ^1^H NMR spectra suggest that THF is coordinated to B(OMgCl)_3_. Based on these results, the synthesized B(OMgCl)_3_ salt has the structure shown in Fig. 6(a), in which THF is coordinated to B(OMgCl)_3_, and a more complicated structure with a disordered network (Fig. 6(b)).

**Figure 7:**
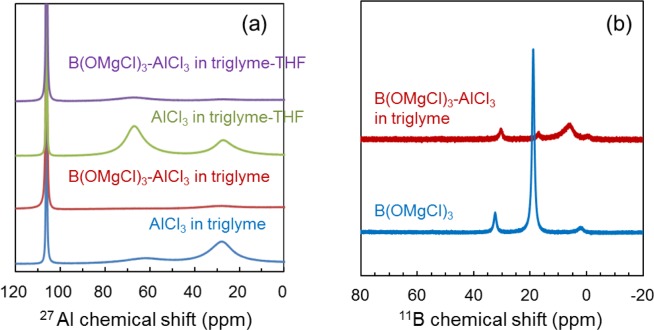
(**a**) ^27^Al NMR spectra of AlCl_3_ in triglyme (blue), B(OMgCl)_3_-AlCl_3_ in triglyme (red), AlCl_3_ in triglyme-THF (50/50 vol%) (green), and B(OMgCl)_3_-AlCl_3_ in triglyme-THF (50:50 vol%) (purple). The concentrations of AlCl_3_ and B(OMgCl)_3_ in all electrolyte solutions are 1.2 and 0.2 M, respectively. (**b**) ^11^B NMR spectra of B(OMgCl)_3_ in CD_3_OD/CD_3_CO_2_D (50/50 vol%) (blue) and B(OMgCl)_3_-AlCl_3_ in triglyme with THF-*d*_8_ (red).

Subsequently, we investigated the Al-coordinated state in B(OMgCl)_3_-based electrolytes by ^27^Al and ^11^B NMR spectroscopy. Figure 7(a) shows the ^27^Al NMR spectra of AlCl_3_/triglyme, B(OMgCl)_3_-AlCl_3_/triglyme, AlCl_3_/triglyme-THF, and B(OMgCl)_3_-AlCl_3_/triglyme-THF measured as neat samples. The peaks in the ^27^Al NMR spectra of both AlCl_3_/triglyme (blue) and AlCl_3_/triglyme-THF (green) without B(OMgCl)_3_ are observed at approximately 106, 62–68, and 27 ppm. On the basis of literature data, the peak at 106 ppm is assigned to the tetrahedral Al species including AlCl_4_^-^ and that at 62–68 ppm to the ether-coordinated AlCl_3_ species^26,45,46^. The peak at approximately 27 ppm is assigned to a positive species, such as glyme-coordinated AlCl_2_^+^^46,47^. AlCl_3_ ionization is expected to occur in triglyme because the dissociation of AlCl_3_ in glyme solvents has been observed, as shown in Eq. (1)^46^:

1 $$2{{\rm{A}}{\rm{l}}{\rm{C}}{\rm{l}}}_{3}\leftrightarrows {{\rm{A}}{\rm{l}}{\rm{C}}{\rm{l}}}_{2}^{+}+{{\rm{A}}{\rm{l}}{\rm{C}}{\rm{l}}}_{4}^{-}.$$

In addition, AlCl_3_ is known to dissociate easily in glyme, but not in THF^46^. Equation (1) is deduced to shift to the left to form AlCl_3_ species (62–68 ppm) in triglyme-THF and to the right to form AlCl_2_^+^ species (27 ppm) in triglyme solution without THF. On the other hand, approximately one peak at about 106 ppm is observed for both B(OMgCl)_3_-AlCl_3_/triglyme and B(OMgCl)_3_-AlCl_3_/triglyme-THF, which are electrolytes containing magnesium salt. The reduced peaks of pentacoordinated AlCl_3_ (62–68 ppm) and AlCl_2_^+^ (27 ppm) in the B(OMgCl)_3_-based electrolyte suggest that these species are bound to the borate anion.

Figure 7(b) displays the ^11^B NMR spectra of a B(OMgCl)_3_-AlCl_3_/triglyme sample diluted with THF-*d*_8_ and B(OMgCl)_3_ sample dissolved in CD_3_OD/CD_3_CO_2_D (50:50 vol%). The ^11^B NMR spectrum of the B(OMgCl)_3_-AlCl_3_/triglyme electrolyte shows an increase in the intensity of the broad peak at around 6 ppm with a decrease in the intensity of the peak at approximately 19 ppm compared with those in the ^11^B NMR spectrum of B(OMgCl)_3_. This suggests that the former peak is due to the BO_3_^3–^ species. Furthermore, these changes in the peak intensities suggest the possibility of rapid exchange of coordination of the borate anion to solvents, Cl^–^, and Al species, among others.

**Figure 8:**
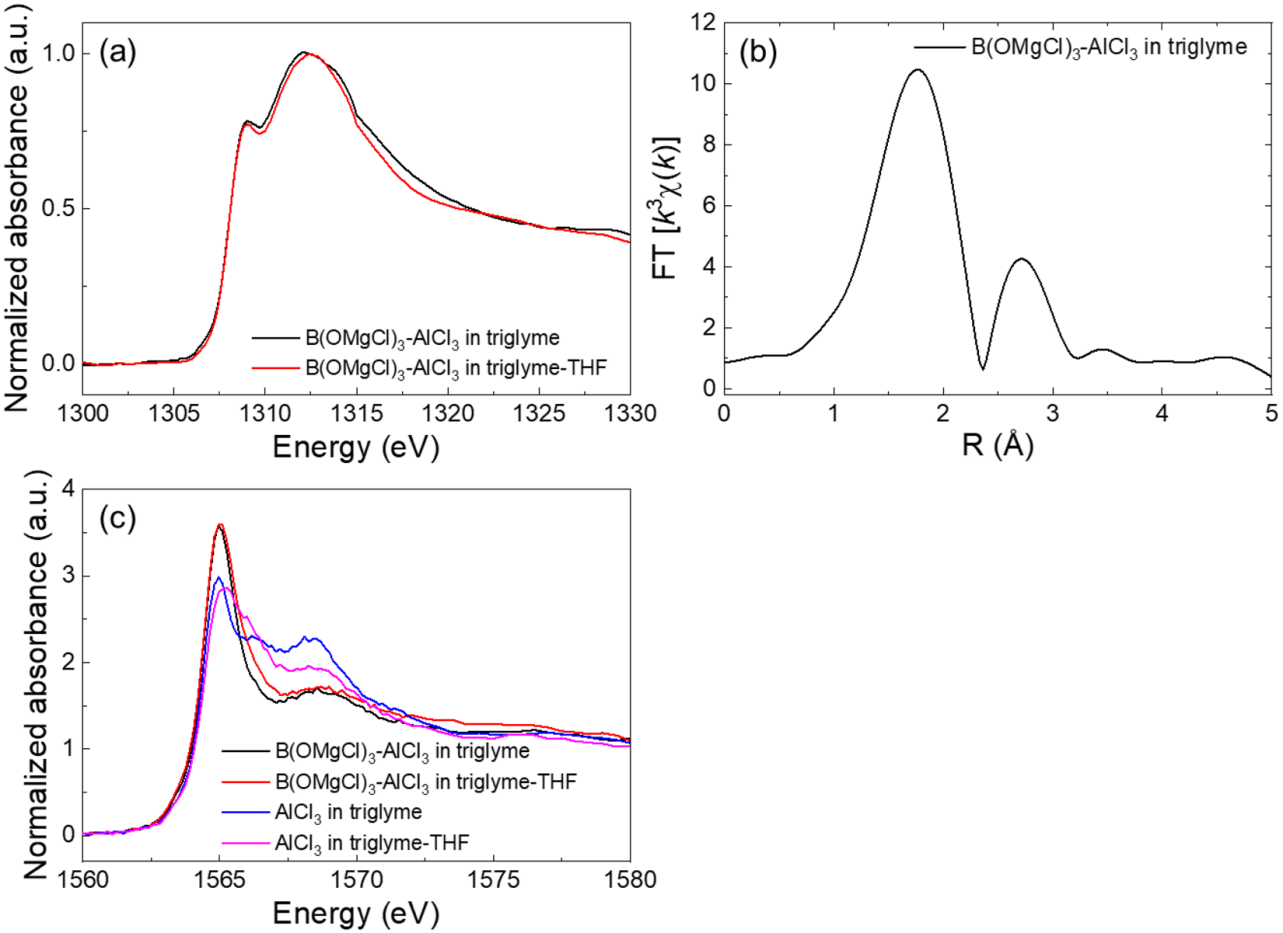
(**a**) Mg *K*-edge XANES spectra of B(OMgCl)_3_-AlCl_3_ in triglyme (black) and B(OMgCl)_3_-AlCl_3_ in triglyme-THF (red) and (**b**) Fourier transform of the EXAFS function of the former. (**c**) Al *K*-edge XANES spectra of B(OMgCl)_3_-AlCl_3_ in triglyme (black), B(OMgCl)_3_-AlCl_3_ in triglyme-THF (red), AlCl_3_ in triglyme (blue), and AlCl_3_ in triglyme-THF (pink).

The B(OMgCl)_3_-based electrolytes were further characterized in terms of electronic and local structures. Figure 8(a) shows the Mg *K*-edge X-ray absorption near edge structure (XANES) spectra of the B(OMgCl)_3_-based electrolytes in triglyme and triglyme-THF. The two main peaks are observed at ~1309 and ~1312 eV with a shoulder at around 1315 eV. The peak at approximately 1309 eV is attributed to either [Mg_2_(μ-Cl)_2_]^2+^ ^48^ or [Mg_2_(μ-Cl)_3_]^+^ species^49^. The position of the main edge at 1312–1315 eV is related to the hexacoordinated solvated structure^48,50^. Figure 8(b) shows the Fourier transform of the Mg *K*-edge extended X-ray absorption fine structure (EXAFS) oscillation of the B(OMgCl)_3_-based electrolyte in triglyme. The Fourier transform for this electrolyte is closest to those for Mg(ClO_4_)_2_/H_2_O^48^ and [Mg_2_(μ-Cl)_3_·6(THF)]^+^ under applied voltage on the Mg electrode^49^. The peak at around 1.8 Å is expected to contain information about the Mg–O pair. On the other hand, it is unclear whether the peak at 2.6 Å is the scattering peak of the Mg–Mg pair or the peak due to the Mg–Cl pair weakened by triglyme. However, the main edge at 1312–1315 eV in the XANES spectrum and the large peak at 2.6 Å in the EXAFS spectrum indicate that the B(OMgCl)_3_-based electrolytes include more electrochemically positive Mg ion than [Mg_2_(μ-Cl)_3_]^+^.

Figure 8(c) represents the Al *K*-edge XANES spectra of the four electrolytes, which are AlCl_3_ with or without B(OMgCl)_3_ dissolved in triglyme or triglyme-THF. Both AlCl_3_ electrolytes without B(OMgCl)_3_ in triglyme (blue) and triglyme-THF (pink) show two edges located at 1565 (with a shoulder at around 1566 eV) and 1568 eV. The shoulder at 1566 eV is smaller and the peak at 1568 eV is larger for AlCl_3_/triglyme compared with those for AlCl_3_/triglyme-THF. These results are consistent with those of ^27^Al NMR showing a smaller peak at 62–68 ppm (due to AlCl_3_ species) and larger peak at 27 ppm (due to AlCl_2_^+^ species) for AlCl_3_/triglyme compared with those for AlCl_3_/triglyme-THF. The Al *K*-edge XANES spectra of the AlCl_3_ electrolytes with B(OMgCl)_3_ show distinctly different absorption curves from those of the AlCl_3_ electrolytes without B(OMgCl)_3_ in both triglyme and triglyme-THF. The increase of the edge at 1565 eV with the decrease of both the shoulder at around 1566 eV and edge at 1568 eV are consistent with the increase of the amount of AlCl_4_^−^ and decrease of the amounts of both AlCl_3_ and AlCl_2_^+^ observed in the ^27^Al-NMR measurement. The edge at 1565 eV, shoulder at around 1566 eV, and edge at 1568 eV in the Al *K*-edge XANES spectra are deduced to be related to AlCl_4_^−^, AlCl_3_, and AlCl_2_^+^, respectively.

The B(OMgCl)_3_-based electrolytes are expected to not cause the corrosive behavior. According to a previous study using MgCl_2_/AlCl_3_ electrolytes, the AlCl_2_^+^ species cause Mg corrosion with Al cementation^51^. However, our ^27^Al NMR analysis (Fig. 7(a)) indicated that the B(OMgCl)_3_ electrolytes do not contain AlCl_2_^+^. Other detected species were solvated [Mg_2_(μ-Cl)_3_]^+^, glyme-solvated Mg^2+^, and AlCl_4_^−^, which reportedly do not cause corrosions.

The results above provide information on the chemical species in the B(OMgCl)_3_-based electrolytes, which can explain the origin of the low Coulombic efficiency. In the case of MACC electrolytes, Mg corrosion with Al cementation caused by AlCl_2_^+^ reduces the Coulombic efficiency^51^. The Coulombic efficiency gradually improves during the CV cycles, which is related to the presence of Al species^27^. However, due to the absence of AlCl_2_^+^ and no improvement in Coulombic efficiency upon CV cycling in the B(OMgCl)_3_-based electrolytes, we conclude that the low Coulombic efficiency observed herein is originated by a different mechanism. Energy-dispersive X-ray spectroscopic analysis of the plated Mg in this study also detected the presence of Al, which can disturb the Mg stripping. The enlarged CV profiles from Fig. 3(a) are provided as Figure S3 in the supporting information, in which the oxidation peaks are not related to Mg stripping but Al stripping. Based on the results of XAFS and NMR, the main species in the electrolytes are solvated [Mg_2_(μ-Cl)_3_]^+^, glyme-solvated Mg^2+^, and AlCl_4_^−^. The presence of AlCl_4_^−^ potentially leads to the following chemical equilibrium^52^:

2 $$2{{\rm{A}}{\rm{l}}{\rm{C}}{\rm{l}}}_{4}^{-}\leftrightarrows {{\rm{A}}{\rm{l}}}_{2}{{\rm{C}}{\rm{l}}}_{7}^{-}+{{\rm{C}}{\rm{l}}}^{-}$$

Compared with conventional MACC electrolytes, the B(OMgCl)_3_-based electrolytes contain excess AlCl_3_ during preparation (Table S1), which drives the equilibrium to the right, resulting in Al plating on the electrode^53^:

3 $$4{{\rm{Al}}}_{2}{{\rm{Cl}}}_{7}^{-}+3{{\rm{e}}}^{-}\to {\rm{Al}}+7{{\rm{AlCl}}}_{4}^{-}$$

This trend is supported by the local structural change observed by Al *K*-edge EXAFS, as shown in Figure S4. The first neighbor shell of Al in the B(OMgCl)_3_-based electrolytes is expanded compared with that of AlCl_4_^−^, which shows good agreement with the literature^52^. However, the ^27^Al NMR analysis of the B(OMgCl)_3_-based electrolytes does not clearly detect the Al_2_Cl_7_^−^ species, which should be observed at 92 ppm. This result implies the low concentration of Al_2_Cl_7_^−^ despite the influence of Al plating. AlCl_3_ concentration must be optimized to improve Coulombic efficiency.

**Figure 9:**
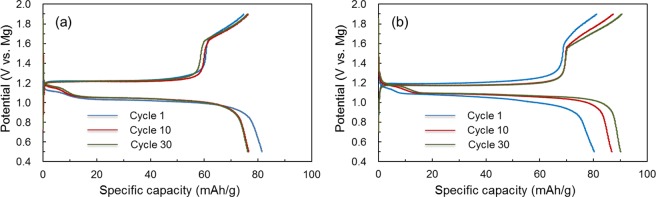
Galvanostatic charge-discharge profiles of a coin-type cell with Mo_6_S_8_ Chevrel phase cathode and AZ31 anode at C/50-rate (based on the mass of Mo_6_S_8_) with the B(OMgCl)_3_-based electrolyte in (**a**) triglyme and (**b**) triglyme-THF.

We have confirmed the high chemical and long-term stability of the B(OMgCl)_3_-based electrolytes and have characterized these electrolytes. Finally, the application of these electrolytes to magnesium rechargeable battery was examined. Figure 9(a,b) represent the galvanostatic charge-discharge profiles of the Mo_6_S_8_/AZ31 coin-type cells with B(OMgCl)_3_-AlCl_3_/triglyme and B(OMgCl)_3_-AlCl_3_/triglyme-THF. Both profiles show plateaus at ~1.05 and ~1.20 V and reversible charge/discharge cycles at a capacity of approximately 80 mAh/g for 30 cycles. The profile of the cell with the B(OMgCl)_3_-AlCl_3_/triglyme-THF electrolyte shows a slightly smaller gap between the charge-discharge plateaus compared with that of the cell with the B(OMgCl)_3_-AlCl_3_/triglyme electrolyte. These results verify that, with B(OMgCl)_3_-based electrolytes, the charge-discharge cycles are repeated on cells with a Chevrel phase cathode and magnesium anode. This indicates that Mg^2+^ insertion/de-insertion into/from the Chevrel phase with Mg plating/stripping proceeds repeatedly. In addition, it also confirms that SUS can be used with a B(OMgCl)_3_-based electrolyte up to 1.9 V vs. Mg. The B(OMgCl)_3_-AlCl_3_/triglyme electrolyte can be used in higher-voltage cathodes (up to 2.1 V vs. Mg)^54^, implying that this electrolyte is useful for the development of novel cathode materials.

## Conclusions

In this study, three novel electrolytes based on Ph_3_COMgCl, Ph_3_SiOMgCl, and B(OMgCl)_3_ were successfully prepared, and the electrochemical properties were investigated. The Ph_3_COMgCl-based electrolyte showed higher anodic stability than the Ph_3_SiOMgCl-based electrolyte, and the B(OMgCl)_3_-based electrolyte showed the highest anodic stability among the three electrolytes. Moreover, the latter was chemically stable, which was ascribed to the Lewis acidic character of boron in B(OMgCl)_3_. Analyses of B(OMgCl)_3_ showed that the Grignard reagent mainly reacted with B(OH)_3_ as a Brønsted acid and revealed that the BO_3_^3−^ unit derived from B(OH)_3_ remained in B(OMgCl)_3_. In addition, the electrochemically positive Mg^2+^ ion is the main species present. Charge-discharge measurements performed for the B(OMgCl)_3_-based electrolyte, a Mo_6_S_8_ Chevrel phase cathode, and an AZ31 anode were indicative of a reversible charge-discharge capacity, suggesting that hardly any side reaction occurred inside SUS cells with the B(OMgCl)_3_-based electrolyte at 1.9 V. Although the B(OMgCl)_3_-based electrolyte did not show high Coulombic efficiency, it showed high anodic stability and can therefore be used in the development of positive electrodes for magnesium batteries.

## Supplementary information

Supplementary Information.
