# Supplementary material for: Improved Non-Grignard Electrolyte Based on Magnesium Borate Trichloride for Rechargeable Magnesium Batteries
Source: Sci Rep. 2020 Apr 30;10:7362. doi: 10.1038/s41598-020-64085-2 (PMC7193642; doi:10.1038/s41598-020-64085-2)
Supplement: Supplementary file 1 — Supplementary Information. [file 41598_2020_64085_MOESM1_ESM.pdf]

Supporting Information for

Improved Non-Grignard Electrolyte Based on Magnesium Borate Trichloride for Rechargeable Magnesium Batteries

Kazuhiko Sato[1], *Goro Mori*[1], *Takahiro Kiyosu*[1], *Toyonari Yaji*[2], *Koji Nakanishi*[2], *Toshiaki Ohta*[2], *Kuniaki Okamoto*[1], *Yuki Orikasa*[*3]

[1]FUJIFILM Wako Pure Chemical Corporation, 1633 Matoba, Kawagoe, Saitama 350-1101, Japan

[2]Synchrotron Radiation Center, Ritsumeikan University, 1-1-1 Nojihigashi, Kusatsu, Shiga 525-8577, Japan

[3]Department of Applied Chemistry, College of Life Sciences, Ritsumeikan University, 1-1-1 Nojihigashi, Kusatsu, Shiga 525-8577, Japan

General Methods

Benzophenone, boric acid, and aluminum chloride were purchased from FUJIFILM Wako Pure Chemical Corporation and used as received. Tetrahydrofuran (THF) and diisopropyl ether (IPE) were purchased in anhydrous form from FUJIFILM Wako Pure Chemical Corporation and used as received. Triglyme was purchased from FUJIFILM Wako Pure Chemical Corporation and used after distillation. Triphenylsilanol, 2 M PhMgCl in THF, and 2 M EtMgCl in THF were purchased from Tokyo Chemical Industry Co., Ltd. and used as received.

Synthesis of magnesium salts

Triphenylmethoxymagnesium chloride ($Ph_3COMgCl$)

A 2 M solution of PhMgCl in THF (20 mL, 40 mmol) was dropwise added to a solution of benzophenone (7.29 g, 40 mmol) in THF (20 mL) at 0 °C, and the reaction mixture was stirred at room temperature (RT) for 4 h under an atmosphere of dry argon. The resulting white precipitate was filtered off, washed with THF, and vacuum-dried at 60 °C to afford $Ph_3COMgCl$ coordinated to THF as a white solid.

Triphenylsiloxymagnesium chloride ($Ph_3SiOMgCl$)

A 2 M solution of PhMgCl in THF (20 mL, 40 mmol) was dropwise added to a solution of triphenylsilanol (11.1 g, 40 mmol) in THF (20 mL) at 0 °C, and the reaction mixture was stirred at RT for 1 h under an atmosphere of dry argon. The resulting suspension was concentrated under reduced pressure and treated with IPE (70 mL). The produced white solid was filtered off, washed with IPE, and vacuum-dried at 60 °C to afford $Ph_3SiOMgCl$ coordinated to THF as a white solid.

Boric acid magnesium chloride ($B(OMgCl)_3$)

A 2 M solution of EtMgCl in THF (30 mL, 60 mmol) was dropwise added to a suspension of boric acid (1.24 g, 20 mmol) in THF (10 mL) at 0 °C, and the reaction mixture was stirred at RT for 1 h under an atmosphere of dry argon. The resulting suspension was concentrated under reduced pressure and treated with IPE (40 mL). The

resulting white solid was filtered off, washed with IPE, and vacuum-dried at 60 °C to afford $B(OMgCl)_3$ coordinated to THF as a white solid.

Table S1. Summary of electrolyte preparation methods.

Electrolyte	Mg salt (mmol)	Triglyme (mL)	$AlCl_3$ (mmol)	conditions	Additional solvent[1]
$Ph_3COMgCl$-based electrolyte in triglyme	5	20	5	50 °C, 5 min	–
$Ph_3SiOMgCl$-based electrolyte in triglyme	5	20	5	50 °C, 10 min	–
$B(OMgCl)_3$-based electrolyte in triglyme	4	10	24	80 °C, 3 d	10 mL triglyme
$B(OMgCl)_3$-based electrolyte in triglyme-THF	4	10	24	80 °C, 3 d	10 mL THF

[1]Only added to $B(OMgCl)_3$-based electrolytes after the reaction.

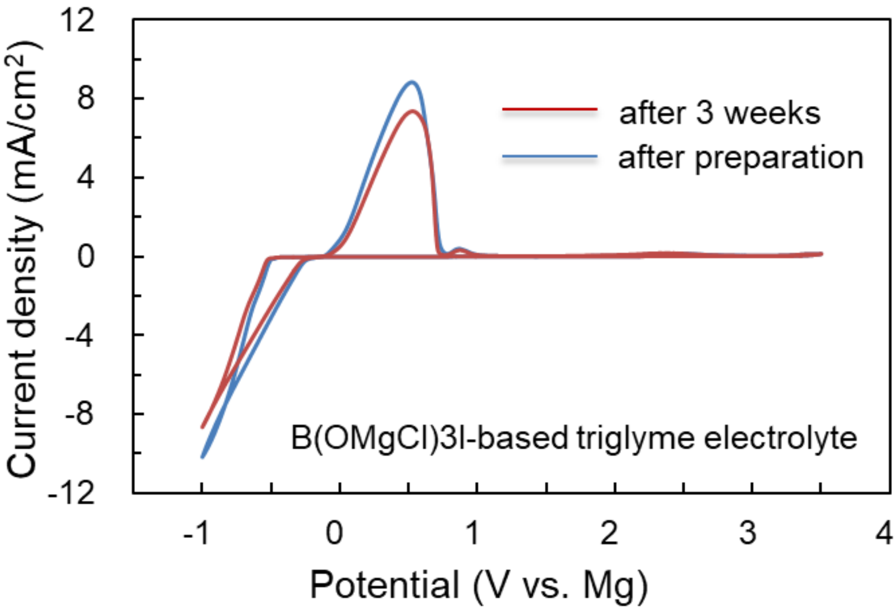

Figure S1. Cyclic voltammograms at the 100th cycle measured at a scan rate of 5 mV/s at 20 °C. The blue and red curves represent the first measurement and measurement after three weeks, respectively, for the $B(OMgCl)_3$-based triglyme electrolyte.

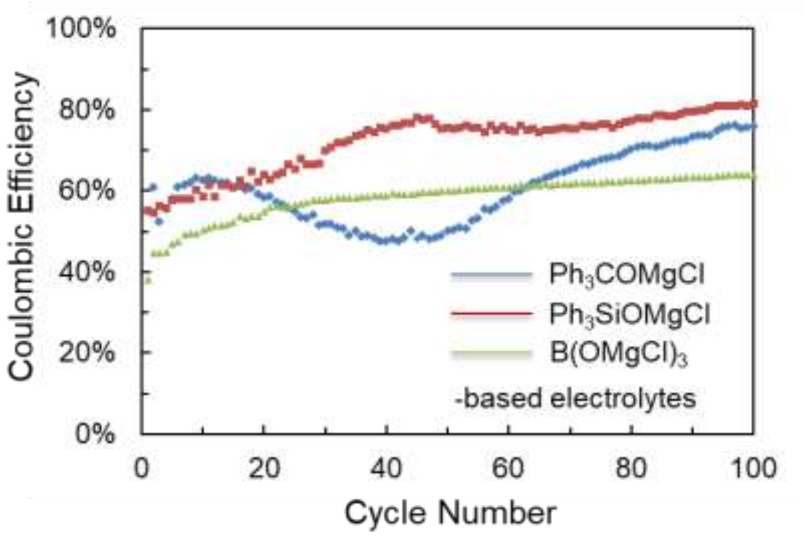

Figure S2. Changes in Coulombic efficiency of $Ph_3COMgCl$- (blue), $Ph_3SiOMgCl$- (red), and $B(OMgCl)_3$-based (green) electrolytes in the Cyclic voltammograms at a scan rate of 5 mV/s at 20 °C

COMPUTATIONAL DETAILS AND CALCULATIONS

Quantum chemical calculations were performed by the B3LYP hybrid density functional method (Becke's three-parameter functional using the LYP correlation functional) using the Gaussian 09W software package. Geometry optimization and frequency analysis were carried out by restricted and unrestricted density functional theory (DFT) calculations with the 6-31+G(d,p) basis set. All optimized structures were characterized to be true local energy minima on the potential energy surface without imaginary frequencies. The sum of electronic and zero-point energies (E_0), calculated from the sum of electronic energy (E_{elec}) and a zero-point energy (ZPE) correction without scaling, was used for energy comparison. Ph_3COH, Ph_3SiOH, and $B(OH)_3$ were used as simple models to discuss the strengths of C–O (in $Ph_3COMgCl$), Si–O (in $Ph_3SiOMgCl$), and B–O (in $B(OMgCl)_3$) bonds. Calculations were also performed for Me_3COH and Me_3SiOH to enable comparison. Depending on whether ionic or radical dissociation was assumed, the optimized structures of ions/radicals and their energies (E_0) were obtained by restricted and unrestricted DFT calculations, respectively. Table S2 shows the values of E_{elec}, ZPE, and E_0 obtained for optimized structures.

Table S2. E_{elec}, ZPE, and E_0 values obtained for each optimized structure.

Species	Method/basis set	Net electric charge	Spin multiplicity	E_{elec} (Hartree)	ZPE (Hartree)	E_0 (kcal/mol)
OH^-	RB3LYP/6-31+G(d,p)	−1	1	−75.803435	0.008554	−47562.046
Me_3C^+		1	1	−157.568782	0.116397	−98802.946
Me_3Si^+		1	1	−409.000004	0.109259	−256583.03
Ph_3C^+		1	1	−732.865412	0.280439	−459704.4
Ph_3Si^+		1	1	−984.265171	0.274516	−617463.98
$(HO)_2B^+$		1	1	−176.291030	0.029864	−110605.64
OH•	UB3LYP/6-31+G(d,p)	0	2	−75.739015	0.008438	−47521.694
Me_3C•		0	2	−157.816747	0.116355	−98958.573
Me_3Si•		0	2	−409.231825	0.109504	−256728.35
Ph_3C•		0	2	−733.083298	0.277754	−459842.81
Ph_3Si•		0	2	−984.468241	0.272637	−617592.58
$(HO)_2B$•		0	2	−176.543636	0.030963	−110763.47
Me_3COH	RB3LYP/6-31+G(d,p)	0	1	−233.701725	0.135292	−146565.27
Me_3SiOH		0	1	−485.165676	0.124416	−304368.24
Ph_3COH		0	1	−808.927672	0.294213	−507425.58
Ph_3SiOH		0	1	−1060.396628	0.287289	−665229.21
$(HO)_2BOH$		0	1	−252.516153	0.048860	−158425.75

Table S3 shows the reaction equations and calculated energy difference (ΔE_0) between the E_0 of each compound and the sum of E_0 of two chemical species generated by bond dissociation. The ΔE_0 values for X–O bonds (X = C, Si, B) calculated by RB3LYP/6-31+G(d,p) and UB3LYP/6-31+G(d,p) methods increased in the order of Ph_3C–OH < Me_3C–OH < Ph_3Si–OH < Me_3Si–OH < $(HO)_2B$–OH, which suggested that C–O bonds are weaker than Si–O and B–O ones. The above trend was identical to that predicted by the hard and soft acid and base model, which suggested that the strength of X–O bonds is influenced by the related molecular orbitals. [1-3] Moreover, the calculated bond strength of Ph_3C–OH was lower than that of Me_3C–OH by 41.1 kcal/mol for the restricted method and by 23.9 kcal/mol for the unrestricted method, while the corresponding decreases observed upon going from Me_3Si–OH to Ph_3Si–OH equaled 20.0 and 2.3 kcal/mol, respectively. This result suggested that the C–O bond was more strongly weakened by Ph substituents than the Si–O bond.

Table S3. Reaction equations and calculated energy difference (ΔE_0) between the E_0 of each compound and the sum of E_0 values of the two chemical species generated by bond dissociation.

**Line**	**Equation**	**ΔE_0 (kcal/mol)**
**1**	Me_3C–OH → Me_3C^+ + $OH^–$	200.3
**2**	Me_3Si–OH → Me_3Si^+ + $OH^–$	223.2
**3**	Ph_3C–OH → Ph_3C^+ + $OH^–$	159.1
**4**	Ph_3Si–OH → Ph_3Si^+ + $OH^–$	203.2
**5**	$(HO)_2B$–OH → $(HO)_2B^+$ + $OH^–$	258.1
**6**	Me_3C–OH → Me_3C• + OH•	85.0
**7**	Me_3Si–OH → Me_3Si• + OH•	118.2
**8**	Ph_3C–OH → Ph_3C• + OH•	61.1
**9**	Ph_3Si–OH → Ph_3Si• + OH•	114.9
**10**	$(HO)_2B$–OH → $(HO)_2B$• + OH•	140.6

Table S4 shows the X–O (X = C, Si, B) bond lengths of optimized structures, demonstrating that the bond of Ph_3C–OH (1.44449 Å) was longer than that of Me_3C–OH (1.44221 Å), while that of Ph_3Si–OH (1.68165 Å) was shorter than that of Me_3Si–OH (1.68681 Å). This structural change suggested that the C–O bond was weakened by change of substituent from Me to Ph, and the above results were used to explain the chemical stabilities of electrolytes in the present paper.

Table S4. X–O (X = C, Si, B) bond lengths of optimized structures.

**Compound**	**Bond length (Å)**
**Me_3C-OH**	1.44421
**Me_3Si-OH**	1.68681
**Ph_3C-OH**	1.44449
**Ph_3Si-OH**	1.68165
**$(HO)_2B$-OH**	1.37219

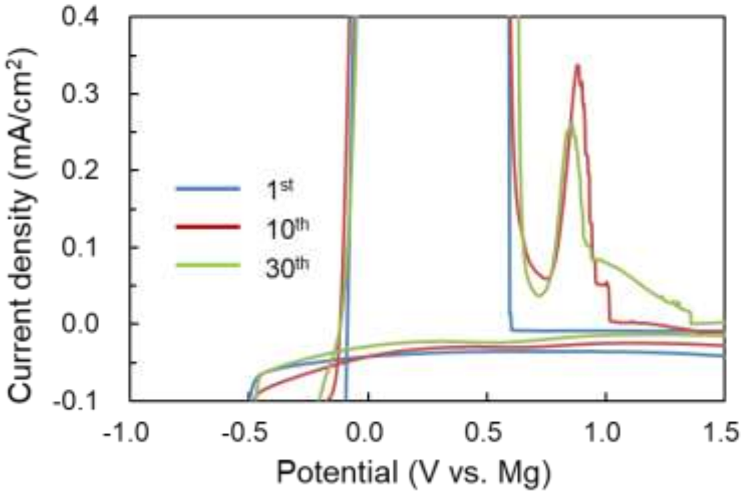

Figure S3. The enlarged figure from Fig. 3 (a) in the manuscript. Cyclic voltammogram of the $B(OMgCl)_3$-based electrolyte in triglyme measured at a scan rate of 5 mV/s at 20 °C. The oxidation peaks of 0.8-0.9V vs Mg are observed in 10 cycle (red line) and 30 cycle (green line), although no oxidation peak 0.8-0.9V vs Mg is observed in the first cycle (blue line). Rough estimation from the standard electrode potentials, this oxidation potential is approximately 0.1-0.2 V vs Al, which implies the presence of Al metal.

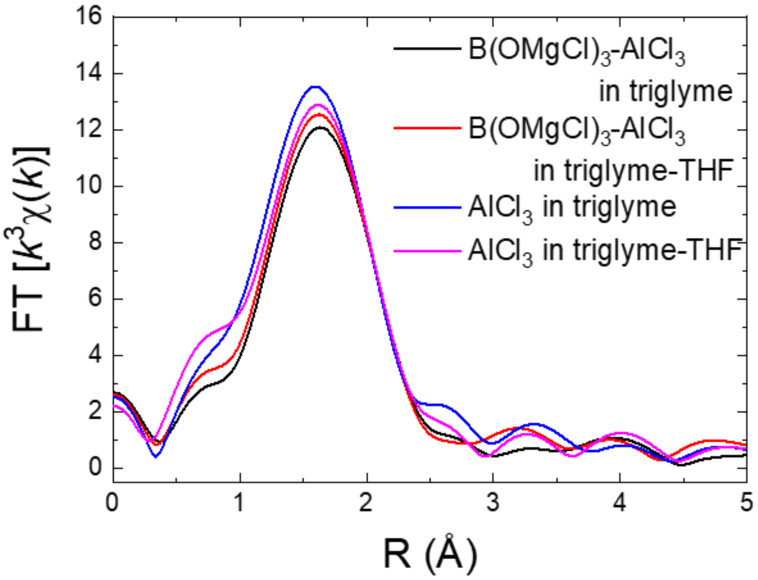

Figure S4. Fourier transform of the EXAFS function at Al *K*-edge of $B(OMgCl)_3$-$AlCl_3$ in triglyme (black), $B(OMgCl)_3$-$AlCl_3$ in triglyme-THF (red), $AlCl_3$ in triglyme (blue), and $AlCl_3$ in triglyme-THF (pink).

References:

1 Ho, T.-L. Hard soft acids bases (HSAB) principle and organic chemistry. *Chem. Rev.* **75**, 1-20, doi:10.1021/cr60293a001 (1975).

2 Klopman, G. Chemical reactivity and the concept of charge- and frontier-controlled reactions. *J. Am. Chem. Soc.* **90**, 223-234, doi:10.1021/ja01004a002 (1968).

3 Hudson, R. F. & Klopman, G. A general perturbation treatment of chemical reactivity. *Tetrahedron Lett.* **8**, 1103-1108, doi:10.1016/S0040-4039(00)90645-2 (1967).

4 Stafford, G. R., Tsuda, T. & Hussey, C. L. The Structure of Electrodeposited Aluminum Alloys from Chloroaluminate Ionic Liquids: Let's Not Ignore the Temperature. *ECS Transactions* **64**, 535-547, doi:10.1149/06404.0535ecst (2014).
